# Rare β-human chorionic gonadotrophin-secreting urothelial carcinoma initially presenting as gynecomastia

**DOI:** 10.1210/jcemcr/luag064

**Published:** 2026-04-24

**Authors:** Gillian M Hoogstraet, Nicola Gathaiya, Paul W Koontz

**Affiliations:** University of Missouri School of Medicine, Springfield Clinical Campus, Springfield, MO 65809, USA; CoxHealth, Adult Medicine and Endocrinology Specialists, Springfield, MO 65807, USA; CoxHealth, Pathology Services of Springfield, Springfield, MO 65807, USA

**Keywords:** gynecomastia, beta-human chorionic gonadotropin-secreting, urothelial carcinoma, urinary bladder neoplasm, paraneoplastic syndrome

## Abstract

Gynecomastia is a relatively common condition arising from disproportionate concentrations of estrogen and androgen. Most cases of bilateral gynecomastia in men are idiopathic or drug-induced but can rarely arise secondary to malignancy. We present the case of a 57-year-old male who presented with new-onset gynecomastia and recurrent episodes of hematuria. Laboratory testing revealed elevated estradiol, free testosterone, and β-human chorionic gonadotropin (β-hCG) with suppressed luteinizing hormone (LH) and follicle stimulating hormone (FSH), concerning for ectopic β-hCG secretion. Mammography, breast and testicular ultrasonography, and chest computed tomography demonstrated no evidence of breast, testicular, or lung malignancy. Concurrent evaluation of hematuria with cystoscopy identified a 6-cm tumor on the posterior bladder wall. The patient underwent transurethral resection of the bladder tumor and was diagnosed with papillary high-grade T1 urothelial carcinoma. The β-hCG immunohistochemical staining of the tumor was positive, and the patient's β-hCG level was undetectable 1 month postoperatively. A review of the literature revealed there are only a few reports detailing urothelial carcinomas capable of secreting β-hCG. To the best of our knowledge, this is the first case of a β-hCG-secreting papillary T1 urothelial carcinoma initially presenting as gynecomastia.

## Introduction

Gynecomastia arises from excessive benign proliferation of glandular breast tissue in males, presenting clinically as a rubbery or firm mass extending from the retro-areolar region. The reported prevalence of gynecomastia in adult males has been estimated to be as high as 36% to 57%, especially in elderly men [1]. Estrogen stimulates the proliferation of breast tissue while androgens exert an inhibitory effect; thus gynecomastia often develops from estrogen excess or androgen deficiency systemically or in the breast tissue [1, 2]. A cause is identified in less than half of adult cases [3]. Drug-induced gynecomastia is the most commonly recognized etiology, but other causes of new-onset gynecomastia in adult men include cirrhosis, chronic renal disease, breast cancer, hypogonadism, hyperthyroidism, hypothyroidism, hyperprolactinemia, and Cushing disease [1, 3, 4]. Gynecomastia can also arise from tumors that increase serum estrogen concentrations through excess estrogen production, through androgen overproduction with peripheral aromatization to estrogens, or via secretion of β-human chorionic gonadotropin (β-hCG) [1, 2]. The pathogenesis of gynecomastia in adult males with elevated β-hCG arises from the ability of β-hCG to bind LH receptors expressed in the testes, promoting steroidogenesis with disproportionate elevation of estrogen compared to testosterone. Adult males exhibiting gynecomastia should thus undergo thorough evaluation due to possible neoplastic etiology. We present the case of a rare β-hCG-secreting papillary urothelial carcinoma that initially manifested as gynecomastia.

## Case presentation

A 57-year-old male with a past medical history of papillary thyroid carcinoma status post total thyroidectomy, neck dissection, and radioactive iodine therapy was originally evaluated in the endocrinology office for a painful lump in his right breast. The lump had been present for 2 months, had not changed in size, and was accompanied by bilateral nipple tenderness. He denied recent trauma or injury to the breasts. He reported no new medications, supplements, or exposures known to cause gynecomastia. He denied a history of smoking or occupational chemical exposures. There was no family history of breast or testicular cancer. The patient endorsed fatigue but otherwise denied additional symptoms, including loss of muscle mass, changes to libido, weight loss, fevers, and weakness. He experienced an episode of gross hematuria a few months prior to presentation, which was initially suspected to be due to dehydration. A second episode of gross hematuria occurred weeks later with no accompanying dysuria, urinary frequency, urgency, or flank pain.

## Diagnostic assessment

On examination, a small mass near the right nipple with clear nipple discharge was appreciated. Testes were descended bilaterally with no testicular masses, and testicular volume was appropriate at approximately 20 mL bilaterally. Initial laboratory evaluation was significant for elevated estradiol of 11 950 pg/mL [International System of Units (SI): 439 pmol/L] (reference range, ≤ 52.50 pg/mL [SI: ≤ 193 pmol/L]) and mildly elevated free testosterone of 187.3 pg/mL (SI: 649 pmol/L) (reference range, 35.0-155.0 pg/mL [SI: 121-537 pmol/L]). Subsequent testing revealed suppressed luteinizing hormone (LH) and follicle stimulating hormone (FSH) in addition to elevated β-hCG of 18 mIU/mL (SI: 18 IU/L) (reference range, < 5 mIU/mL [SI: < 5 IU/L]). Sex hormone binding globulin (SHBG), corticotropin (ACTH), insulin-like growth factor-1 (IGF-1), thyroid stimulating hormone (TSH), prolactin, and α-fetoprotein were normal (Table 1).

**Table 1.** Summary of laboratory values before and after resection of urothelial carcinoma

*Hormone*	Reference range	Baseline lab values	Repeat preoperative labs	Labs after tumor resection
β−hCG	<5 mlU/mL (<5 IU/L)	**18 mIU/mL (18 IU/L)**	**36 mIU/mL (36 IU/L)**	<5 mIU/mL (<5 IU/L)
Prolactin	2.5-17.4 ng/mL (2.5-17.4 μg/L)	7.6 ng/mL (7.6 μg/L)	2.7 ng/mL (2.7 μg/L)	ND
LH	1.20-10.60 mIU/mL (1.20-10.60 IU/L)	**<0.10 mIU/mL (<0.10 IU/L)**	ND	2.7 mIU/mL (2.7 IU/L)
FSH	0.70-10.80 mIU/mL (0.70-10.80 IU/L)	**< 0.30 mIU/mL (<0.30 IU/L)**	ND	4.4 mIU/mL (4.4 IU/L)
Total testosterone	250-1100 ng/dL (8.67-38.2 nmol/L)	790 ng/dL (27.4 nmol/L)	1042 ng/dL (36.1 nmol/L)	336 ng/dL (11.7 nmol/L)
Free testosterone	35.0-155.0 pg/mL (121-537 pmol/L)	**187.3 pg/mL (649 pmol/L)**	ND	43.7 pg/mL (152 pmol/L)
Bioavailable testosterone	110.0-575.0 ng/dL (3.81-19.9 nmol/L)	360.7 ng/dL (12.5 nmol/L)	ND	ND
Estrone	≤ 68 pg/mL (≤ 250 pmol/L)	67 pg/mL (246 pmol/L)	ND	ND
Estriol	≤ 0.18 ng/mL (≤ 0.62 nmol/L)	<0.10 ng/mL (<0.35 nmol/L)	ND	ND
Estradiol	≤ 52.50 pg/mL (≤ 193 pmol/L)	**119.50 pg/mL (439 pmol/L)**	**139.40 pg/mL (511 pmol/L)**	36.90 pg/mL (136 pmol/L)
SHBG	2.47-8.66 μg/mL (22-77 nmol/L)	3.37 μg/mL (30 nmol/L)	ND	ND
TSH	0.358-3.740 μIU/mL (0.358-3.740 mIU/L)	2.150 μIU/mL (2.150 mIU/L)	ND	ND
ACTH	6-50 pg/mL (1.32-11.00 pmol/L)	22 pg/mL (4.8 pmol/L)	ND	ND
Cortisol, serum Am	5-22 μg/dL (138-607 nmol/L)	15.28 μg/dL (421 nmol/L)	ND	ND
IGF-1	50-317 ng/mL (6.55-41.5 nmol/L)	242 ng/mL (31.6 nmol/L)	ND	ND
AFP	<6.1 ng/mL (<6.1 μg/L)	2.7 ng/mL (2.7 μg/L)	ND	ND

Abnormal values shown in bold font. Values in parentheses are International System of Units.

Abbreviations: AFP, α-fetoprotein; β-hCG, β-human chorionic gonadotrophin; ND, no data.

Mammography and breast ultrasound were performed to better characterize the right breast mass and exclude malignancy. There was mild gynecomastia in the area of the right breast mass and in the left breast retro-areolar space on mammography. The mammogram showed no border-forming masses, architectural distortion, or suspicious calcifications. Breast ultrasound corroborated that the right breast mass was consistent gynecomastia. Scrotal ultrasound showed no sonographic evidence of testicular malignancy. Other than mild gynecomastia, no acute findings were present on chest computed tomography (CT) with contrast. Magnetic resonance imaging (MRI) of the pituitary was performed, which showed a normal pituitary gland with no defects or masses and a normal infundibular stalk with no parasellar masses.

The patient was referred to urology for further evaluation of intermittent gross hematuria. Urinalysis was significant for red blood cells > 100 per high-power field and trace protein. Kidney, ureter, and bladder X-ray performed shortly after showed no evidence of nephrolithiasis. Simple hepatic and renal cysts and nonobstructing left renal stones measuring 2.9 mm or less without hydroureteronephrosis were found on abdominopelvic CT. There was no evidence of hepatic, pancreatic, adrenal, or renal malignancy and no lymphadenopathy on imaging. CT urogram revealed semi-circumferential thickening of the right anterolateral urinary bladder wall with adjacent fat stranding suspicious for malignancy. Cystoscopy showed a 6-cm papillary tumor on the posterior bladder wall.

## Treatment

**Figure 1.**
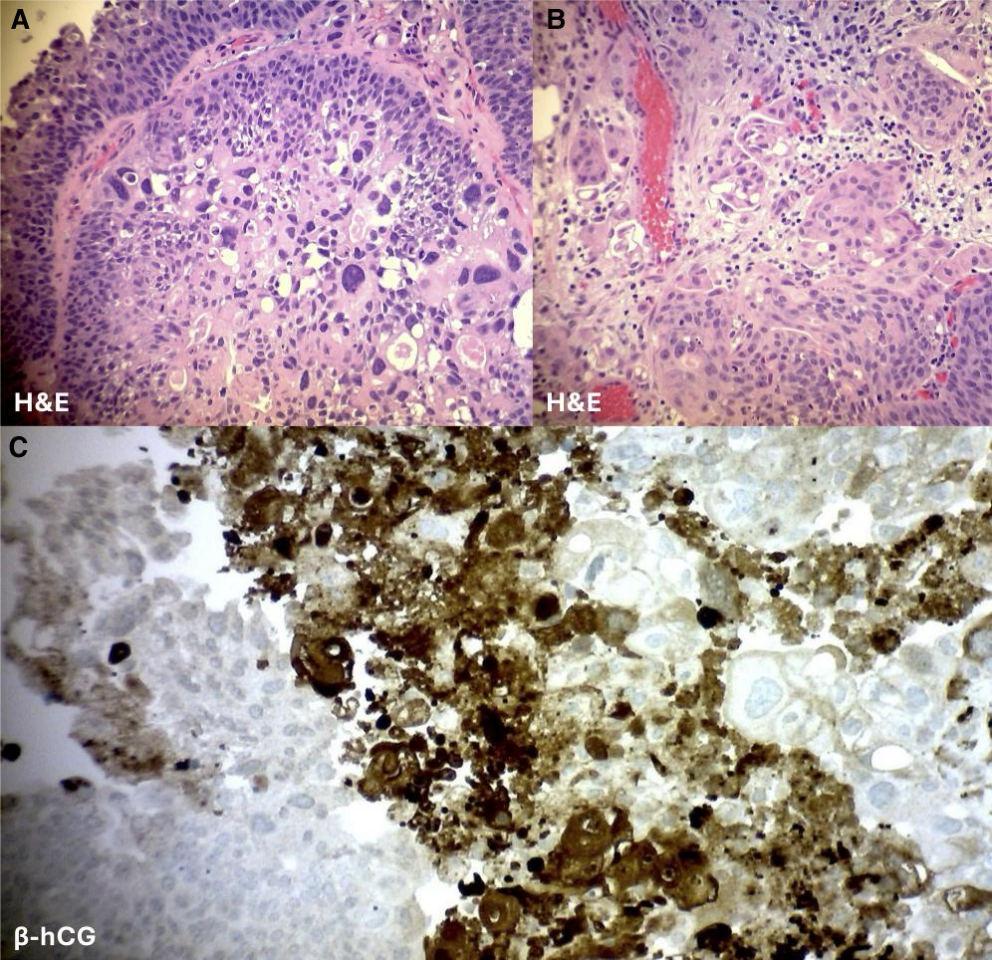
Urothelial carcinoma pathology. (A) Hematoxylin and eosin-stained sections show focal collections of pleomorphic tumor cells, 200×. (B) The urothelial carcinoma demonstrated invasion into the lamina propria, 200×. (C) Pleomorphic tumor cells are positive for β-hCG by immunohistochemical stain, supporting the bladder tumor as the likely source of ectopic β-hCG secretion, 200×. Abbreviation: β-h CG, β-human chorionic gonadotrophin.

The patient underwent transurethral resection of the bladder tumor. Pathology of the tumor was consistent with papillary high-grade T1 urothelial carcinoma with glandular differentiation and lamina propria invasion (Fig. 1A and 1B). No muscularis propria invasion was present on initial pathology or on repeat cystoscopy with biopsy 2 weeks later. There was no obvious residual disease postoperatively. The β-hCG immunohistochemical staining of the bladder tumor demonstrated focal positivity with adequate control (Fig. 1C). The patient is undergoing Bacillus Calmette-Guérin induction therapy to reduce the risk of bladder cancer recurrence.

## Outcome and follow-up

**Figure 2.**
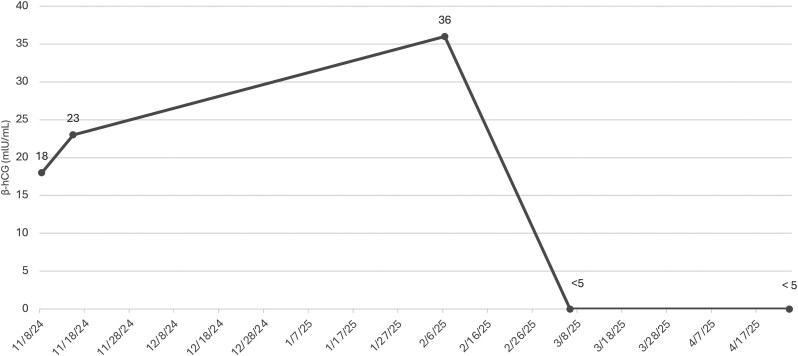
Trend of β-hCG levels during initial evaluation, 1 month postoperatively, and 2 months postoperatively following resection of urothelial carcinoma. Abbreviation: β-h CG, β-human chorionic gonadotrophin.

Laboratory testing 3 weeks postoperatively revealed the patient's β-hCG level was undetectable at less than 5 mIU/mL (Fig. 2). His β-hCG level remained undetectable, and LH, FSH, estradiol, and testosterone levels normalized 2 months postoperatively with no biochemical evidence of disease recurrence (Table 1). The patient had no clinical gynecomastia when evaluated at 6 months postoperatively.

## Discussion

Although rare, neoplasm as an underlying cause of gynecomastia must always be considered. A detailed history should be obtained as part of the initial evaluation, including duration of breast enlargement, sexual function, unintended weight loss, medication history, family history, and concurrent medical conditions. Physical examination is important to differentiate gynecomastia from pseudogynecomastia. Gynecomastia presents as concentric, firm tissue directly beneath the nipple-areolar complex, while pseudogynecomastia will not be firm on palpation. Breast carcinomas are typically unilateral, firm, and outside the areola [4, 5]. Testicular examination in the setting of male gynecomastia may reveal a mass consistent with a human chorionic gonadotrophin-secreting testicular tumor or decreased testicular size potentially secondary to hypogonadism [6, 7].

Laboratory testing should include renal, hepatic, and thyroid function tests to exclude the aforementioned pathologies as well as hormonal testing of total and free testosterone, estrogen, LH, FSH, and prolactin levels [5]. The patient had elevated estradiol and mildly elevated testosterone, as well as suppressed LH and FSH. Due to these findings, β-hCG was tested and found to be elevated. Elevated β-hCG, estradiol, and free/total testosterone in combination with suppressed LH and FSH raised concern that his gynecomastia may be paraneoplastic secondary to ectopic β-hCG production from a testicular/extragonadal germ cell tumor or lung malignancy.

Various imaging modalities can be utilized to evaluate gynecomastia and elevated β-hCG in a male patient. A mammogram and ultrasound were obtained to ensure the right-sided breast mass was indeed consistent with gynecomastia and not suspicious for breast carcinoma. Scrotal ultrasonography found no evidence of a testicular tumor as the source of β-hCG secretion, and MRI brain showed no masses concerning for central nervous system germinoma. Lung cancers have been reported to present with gynecomastia as a paraneoplastic syndrome secondary to their ability to ectopically produce and secrete β-hCG [8, 9]. Chest CT was unrevealing, making a diagnosis of lung cancer unlikely.

Previous reports document pituitary adenomas capable of expressing β-hCG, so a pituitary MRI was performed to determine if a pituitary adenoma was underlying his gynecomastia [10, 11]. However, no abnormalities were identified on MRI. Additionally, labs to identify a functional pituitary adenoma including prolactin, TSH, ACTH, cortisol, and IGF-1 were tested and found to be within normal limits. In combination with suppressed LH and FSH, a pituitary pathology was an unlikely cause of the patient's gynecomastia and elevated β-hCG levels.

Given the concurrent presentation of urothelial carcinoma and elevated β-hCG with gynecomastia, suspicion was high that the bladder tumor may be the source of β-hCG secretion. Following surgical excision of the urothelial carcinoma, the patient's previously elevated β-hCG, LH, and FSH resolved, and testosterone levels normalized. Pathology findings were significant for focal positivity on β-hCG immunohistochemical staining of the bladder tumor. Therefore, the diagnosis of paraneoplastic gynecomastia associated with ectopic production of β-hCG by a high-grade papillary urothelial carcinoma was determined. Consideration could be given for the use of β-hCG as a tumor biomarker to monitor for urothelial cancer recurrence as part of future management.

This case is unique in that it is a rare presentation of a paraneoplastic endocrine effect caused by urothelial carcinoma of the bladder. Highly malignant tumors involving the lungs, breasts, prostate, ovaries, skin, colon, and hematologic malignancies are implicated in the majority of paraneoplastic endocrine syndromes arising from nonendocrine neoplasms. Gynecomastia due to β-hCG production is a less common paraneoplastic effect compared to humoral hypercalcemia of malignancy, syndrome of inappropriate antidiuretic hormone secretion, and Cushing syndrome [12-14]. Furthermore, ectopic β-hCG production resulting in gynecomastia has mainly been reported secondary to small cell lung cancer, bronchial carcinoids, germ cell tumors, and pancreatic neuroendocrine tumors [6-9, 13, 15]. There are only a handful of cases where a urothelial carcinoma was found to be capable of β-hCG production. Caron et al detailed 1 of the first reports of gynecomastia in a 50-year-old male induced by ectopic production of β-hCG by a grade III stage A urothelial carcinoma in 1984 [14]. A 71-year-old female patient was found to have metastatic urothelial carcinoma that tested positive for β-hCG as well as granulocyte-colony stimulating factor and PTH-related peptide [16]. Venyo et al studied the immunohistological expression of β-hCG in 86 urothelial carcinomas and found β-hCG expression was more common in poorly differentiated tumors of higher grade and was associated with inferior outcomes [17]. This study did not, however, investigate the serum β-hCG levels of the patients or clinical presentation. To our knowledge, this is the first reported case of a papillary high-grade T1 urothelial carcinoma capable of β-hCG expression and secretion with subsequent hyperestrogenism manifesting as paraneoplastic gynecomastia.

## Learning points

- Although new-onset gynecomastia in adult men is commonly idiopathic or drug-induced, it warrants thorough evaluation for potential paraneoplastic hormone secretion as an underlying cause.
- Ectopic β-hCG secretion by urothelial carcinomas is a rare clinical phenomenon. These tumors may be underrecognized due to a lack of specific symptoms and an inability to visualize on traditional imaging modalities like CT and MRI.
- For patients with urothelial carcinomas capable of secreting β-hCG, repeat laboratory monitoring of β-hCG levels could serve as a tumor biomarker to evaluate disease progression and response to treatment.

## Contributors

All authors made individual contributions to authorship. G.M.H. was involved in the acquisition of the data and preparation of the manuscript. N.G. supervised the preparation of the manuscript and was involved in the diagnosis, management, and follow-up of the patient. P.W.K. was involved in the diagnosis of the patient, tumor immunostaining, and preparation of histology images. All authors reviewed and approved the final draft of the manuscript.

## Data Availability

Original data generated and analyzed during this study are included in this published article.
